# Synchronized Biventricular Heart Pacing in a Closed-chest Porcine Model based on Wirelessly Powered Leadless Pacemakers

**DOI:** 10.1038/s41598-020-59017-z

**Published:** 2020-02-07

**Authors:** Hongming Lyu, Mathews John, David Burkland, Brian Greet, Allison Post, Aydin Babakhani, Mehdi Razavi

**Affiliations:** 1 Electrical and Computer Engineering Department, University of California Los Angeles, 420 Westwood Plaza, Los Angeles, CA 90095 USA; 2 Texas Heart Institute, 6770 Bertner Avenue, Houston, TX 77030 USA; 3 School of Medicine, Baylor College of Medicine, 1 Baylor Plaza, Houston, TX 77030 USA

**Keywords:** Cardiac device therapy, Electrical and electronic engineering

## Abstract

About 30% of patients with impaired cardiac function have ventricular dyssynchrony and seek cardiac resynchronization therapy (CRT). In this study, we demonstrate synchronized biventricular (BiV) pacing in a leadless fashion by implementing miniaturized and wirelessly powered pacemakers. With their flexible form factors, two pacemakers were implanted epicardially on the right and left ventricles of a porcine model and were inductively powered at 13.56 MHz and 40.68 MHz industrial, scientific, and medical (ISM) bands, respectively. The power consumption of these pacemakers is reduced to µW-level by a novel integrated circuit design, which considerably extends the maximum operating distance. Leadless BiV pacing is demonstrated for the first time in both open-chest and closed-chest porcine settings. The clinical outcomes associated with different interventricular delays are verified through electrophysiologic and hemodynamic responses. The closed-chest pacing only requires the external source power of 0.3 W and 0.8 W at 13.56 MHz and 40.68 MHz, respectively, which leads to specific absorption rates (SARs) 2–3 orders of magnitude lower than the safety regulation limit. This work serves as a basis for future wirelessly powered leadless pacemakers that address various cardiac resynchronization challenges.

## Introduction

Miniaturized implantable medical devices (IMDs), overcoming the physical constraints of conventional devices, provide novel diagnostic and therapeutic solutions in the medical space. Examples include implantable neural interface systems that achieve high spatiotemporal resolution and spatial coverage^1^, microchips capable of wirelessly programmed scheduling of drug delivery for patients with osteoporosis^2^, and a disposable endoscopic video capsule which permits visualization of the gastrointestinal tract by wireless transmission of images^3^. While many remain battery-powered, wireless power transfer techniques can further reduce the form factor and invasiveness of these devices^4–10^.

A conventional cardiac pacemaker consists of a pulse generator and pacing leads positioned using a transvenous approach. Because it contains the battery, the pulse generator is relatively bulky. It is typically placed in the chest or abdomen, with the leads connecting to it on one end and to the myocardium on the other. Approximately 30% of patients with chronic heart failure have electrical dyssynchrony in myocardial activation^11^, and require cardiac resynchronization therapies (CRTs). Existing CRTs rely on the use of multiple intracardiac leads to pace both ventricles in a synchronized sequence^12^. While clinical improvements are generally achieved, six-month nonresponder rates have been reported to be as high as 32–43%^11,13^, which is partially attributed to anatomic constraints of lead positioning^14^. Transvenous leads are also associated with various complications such as lead dislodgement^15^, tricuspid valve dysfunction^16^, and thromboembolism^17^, etc., which would be solved with leadless pacemaker technologies^18–20^.

Miniaturized and wirelessly powered pacemakers are envisioned to be directly implanted at the desired pacing sites. Not only is the need for intravascular leads eliminated, but synchronized and leadless pacing across different chambers becomes feasible, which offers the flexibility in customizing patient-specific CRTs. Prior studies have investigated wirelessly powered single-site cardiac pacing on rodent, rabbit and open-chest porcine models^4,5,21^. This work takes a stride further to demonstrate the synchronization of multiple leadless pacemakers for the first time in a closed-chest porcine model. The improved clinical outcome is verified through both electrophysiologic and hemodynamic studies.

Local energy sources such as energy harvested from the cardiac and lung motions have been exploited as the replacement to batteries^22,23^, but is suspected to be insufficient in its power density^4^. On the contrary, electromagnetic energy in near- and mid-field supplies sufficient power to medical implants^4,24^, as magnetic fields penetrate through tissue as in free space^25^. Utilization of such energy, however, poses two challenges: first, achieving resonant coupling in the low-frequency regime could often result in over-size of the implant due to the associated long wavelength. Second, the magnetic field decays as the inverse cube of distance limiting the maximum operating range^26^. This study seeks to mitigate the first challenge by achieving a relatively miniaturized, flexible and lightweight design to minimize the invasiveness for epicardial implantation^27,28^. For the second, the power consumption of the pacemaker is substantially reduced by customizing a low-power integrated circuit (IC), which alleviates the need for the incident power and, therefore, extends the distance of operation.

In this work, we implement two inductively powered leadless pacemakers operating at 13.56 MHz and 40.68 MHz industrial, scientific, and medical (ISM) bands, respectively. The pacemaker features a µW-level power consumption enabled by a custom low-power IC and is implemented on a flexible polyimide substrate with a dimension of 11 mm × 11 mm. To demonstrate wireless CRT, two such pacemakers were epicardially implanted on the right ventricle (RV) and left ventricle (LV) in a porcine model for synchronized biventricular (BiV) pacing with controllable interventricular offsets. BiV pacing with zero offset achieves a QRS duration and hemodynamic response comparable to those of the intrinsic non-pathologic heartbeat. Closed-chest BiV pacing was further demonstrated with the specific absorption rate (SAR) orders of magnitude lower than the safety regulation limit. This work is the first demonstration of the multisite CRT based on batteryless and leadless pacemakers with improved clinical outcomes in both interventricular synchronization and cardiac stroke volumes. The wireless powering strategy can be applied to various other medical implants as well.

## Results

### System overview

**Figure 1:**
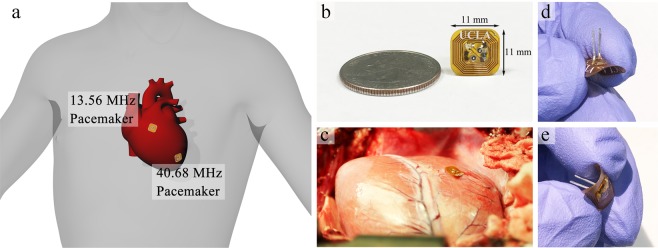
Conceptual illustration of BiV pacing and photos of the proposed pacemaker. (**a**) Illustration of BiV pacing based on the proposed pacemakers. (**b**) Picture of the pacemaker compared with a US quarter. (**c**) Picture of the pacemaker implanted on the heart surface in a porcine model. (**d**,**e**) Pictures showing the flexibility of the pacemaker.

BiV pacing in this work is conceptually illustrated in Fig. 1a. The pacemakers with the flexible design can be directly plunged into the cardiac epicardium. Through resonant frequency selection, two pacemakers can be independently controlled in a BiV pacing setting. The interventricular offsets can be programmed to optimize the clinical outcome according to established clinical practices^14^. The as-fabricated pacemaker is displayed in Fig. 1b. The device features an 11 mm × 11 mm dimension and resides on a 25 µm thick flexible polyimide substrate housing the traces and the inductive coil. Owing to this design, the pacemaker closely attaches to the surface of the heart as shown in Fig. 1c, allowing the natural motion of the heart after implantation. The flexibility of the device is further demonstrated in Fig. 1d,e.

### Pacemaker design

**Figure 2:**
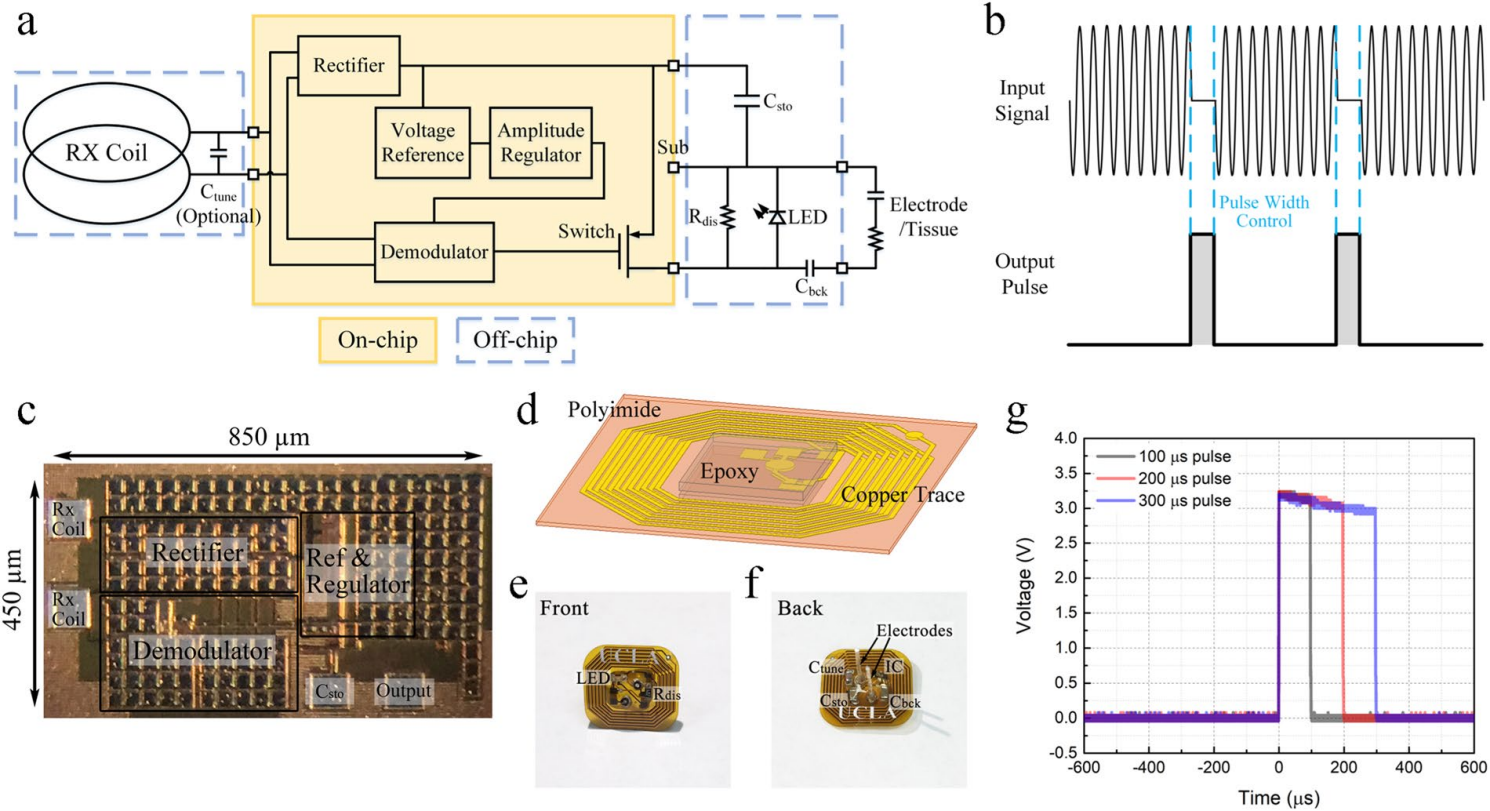
Pacemaker design. (**a**) Block diagram of the pacemaker circuity. (**b**) Conceptual waveforms that illustrate the pulse modulation scheme. (**c**) Picture of the fabricated IC. (**d**) 3D model of the Rx coil. Pictures of the (**e**) front and (**f**) back sides of the pacemaker assembly. (**g**) Stimulation waveforms with 100 µs, 200 µs, and 300 µs pulse width.

Due to their excellent energy-efficiency^29^, most existing implantable pulse generators (IPGs) rely on the voltage-controlled stimulation (VCS) scheme^30^. The proposed pacemaker utilizes the VCS scheme with the core circuitry detailed in ref. ^7^. The block diagram schematic is shown in Fig. 2a. Most of the circuitry is integrated in a complimentary-metal-oxide-semiconductor (CMOS) IC that includes the following main blocks: (1) A rectifier that harvests the incident inductive energy and stores it on a storage capacitor, C_sto_. (2) A voltage reference and an amplitude regulator that regulates the voltage of the stimulations. (3) A demodulator that controls the timing and intensity of the stimulations.

The operation scheme is illustrated in Fig. 2b. The transmitting (Tx) signal involves notches with programmable rates and widths. The IC detects and replicates these notches as the timing of the output stimulations^7^. This method significantly simplifies the circuitry compared to prior arts^31,32^. The voltage of the pulses is regulated, and the pulse width is used to controls the stimulation intensity. As the notches in the Tx signal only constitute a negligible portion timewise, they do not affect the wireless power transfer efficiency.

The IC only consumes a static current consumption of 950 nA. As the supply voltage is about 3.3 V, the static power is merely 3 µW. It also features a very small dimension, a pad-included area of 850 µm × 450 µm as shown in Fig. 2c. The limited number of discrete components include an energy storage capacitor, C_sto_, a DC-block capacitor, C_bck_, for charge-neutralization of the stimulations, and a tuning capacitor, C_tune_, that adjusts the frontend resonant frequency. In addition, this work implements a green light-emitting diode (LED) in the assembly, which helps to illustratively indicate the operation of the device.

The pacemaker resides on a 25 µm thick flexible polyimide substrate that incorporates a power receiving (Rx) coil, as shown in Fig. 2d. The Rx coil features a double-sided design with six turns on both sides. The width and thickness of the copper trace are 7 mils and 1 mil, respectively. The inductance of the Rx coils is about 1.8 µH and the self-resonance frequency (SRF) is approximately at 40.68 MHz ISM band. Adding a C_tune_ of 82 µF in parallel with Rx coil tunes the SRF to be at around 13.56 MHz ISM band. Two pieces of 24 AWG stainless wire are used as the electrodes as well as the anchors for epicardial implantation. The front and back sides of the device are shown in Fig. 2e,f, respectively. Measured output stimulation waveforms with 100 µs, 200 µs, and 300 µs pulse width are shown in Fig. 2g. The weight of each pacemaker is only about 90 mg due to the elimination of batteries.

### Inductive power transfer link

**Figure 3:**
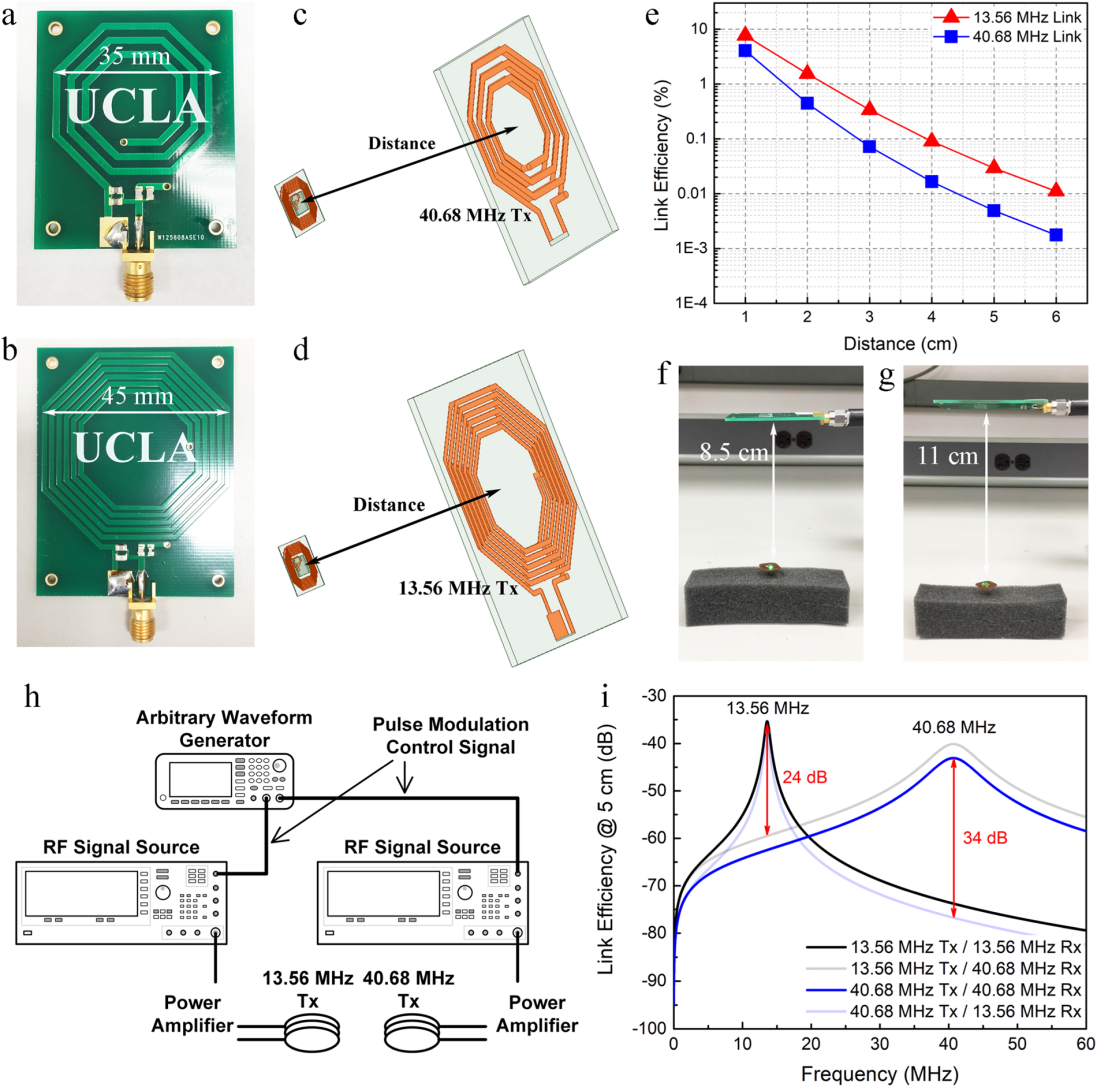
Inductive coupling links. Pictures of the (**a**) 40.68 MHz and (**b**) 13.56 MHz Tx coils. 3D model of the inductive coupling links at (**c**) 40.68 MHz and (**d**) 13.56 MHz bands. (**e**) Simulated link efficiencies as a function of Tx-Rx distance. Pictures that show the maximum operating distance for (**f**) 40.68 MHz and (**g**) 13.56 MHz pacemakers. (**h**) The schematic of the setup that controls the two pacemakers in synchronization with programmable offsets. (**i**) The mutual isolation between the 13.56 MHz and 40.68 MHz channels at 5 cm Tx-Rx separation.

40.68 MHz and 13.56 MHz Tx coils are implemented on FR4 substrates. The 40.68 MHz coil has three turns on both sides with an outer diameter of 35 mm as shown in Fig. 3a. The backside of the 40.68 MHz coil is shown in Supplementary Fig. S1a. The 13.56 MHz coil has a slightly larger dimension with six turns on both sides and an outer diameter of 45 mm, as shown in Fig. 3b. Its backside is shown in Supplementary Fig. S1b. The link efficiency is simulated with Maxwell (Ansys, Inc) and Simplorer (Ansys, Inc). Figure 3c,d demonstrate the 3D models for the 40.68 MHz and 13.56 MHz links, respectively. The link efficiency as a function of Tx-Rx distance is shown in Fig. 3e. The 13.56 MHz shows a slower decay because of the slightly larger Tx coil. Both wireless power transfer links are validated in benchtop tests. With 1 W Tx power, 40.68 MHz and 13.56 MHz links render the maximum operating distance of 8.5 cm and 11 cm as shown in Fig. 3f,g, respectively. It shows that while the efficiency of near-field inductive coupling systems decays according to the inverse-cubic relationship, µW-level wirelessly powered implants can still obtain a considerable range of operation. It is also worth noting that since the magnetic field penetrates through tissues equally as in the free air, biological layers in the link do not affect the coupling efficiency.

The schematic in Fig. 3h illustrates the setup for the synchronization of the two pacemakers in this study. An arbitrary waveform generator outputs two synchronized duty-cycled pulse signals that control two following signal sources via pulse modulation. The two signal sources generate RF signals at 13.56 MHz and 40.68 MHz ISM bands, respectively, which inductively couple to the corresponding pacemakers. Assuming Tx-Rx distance of 5 cm, the isolation between the 13.56 MHz and 40.68 MHz channels is simulated as shown in Fig. 3i. At 13.56 MHz, the selectivity of the 13.56 MHz pacemaker over the 40.68 MHz pacemaker is 24 dB (251x). Likely, at 40.68 MHz, the selectivity of the 40.68 MHz pacemaker over the 13.56 MHz pacemaker is 34 dB (2512x). The independent control of the 13.56 MHz and 40.68 MHz pacemakers placed at the proximity is verified and shown in the photos in Supplementary Fig. S2.

### Open-chest pacing

**Figure 4:**
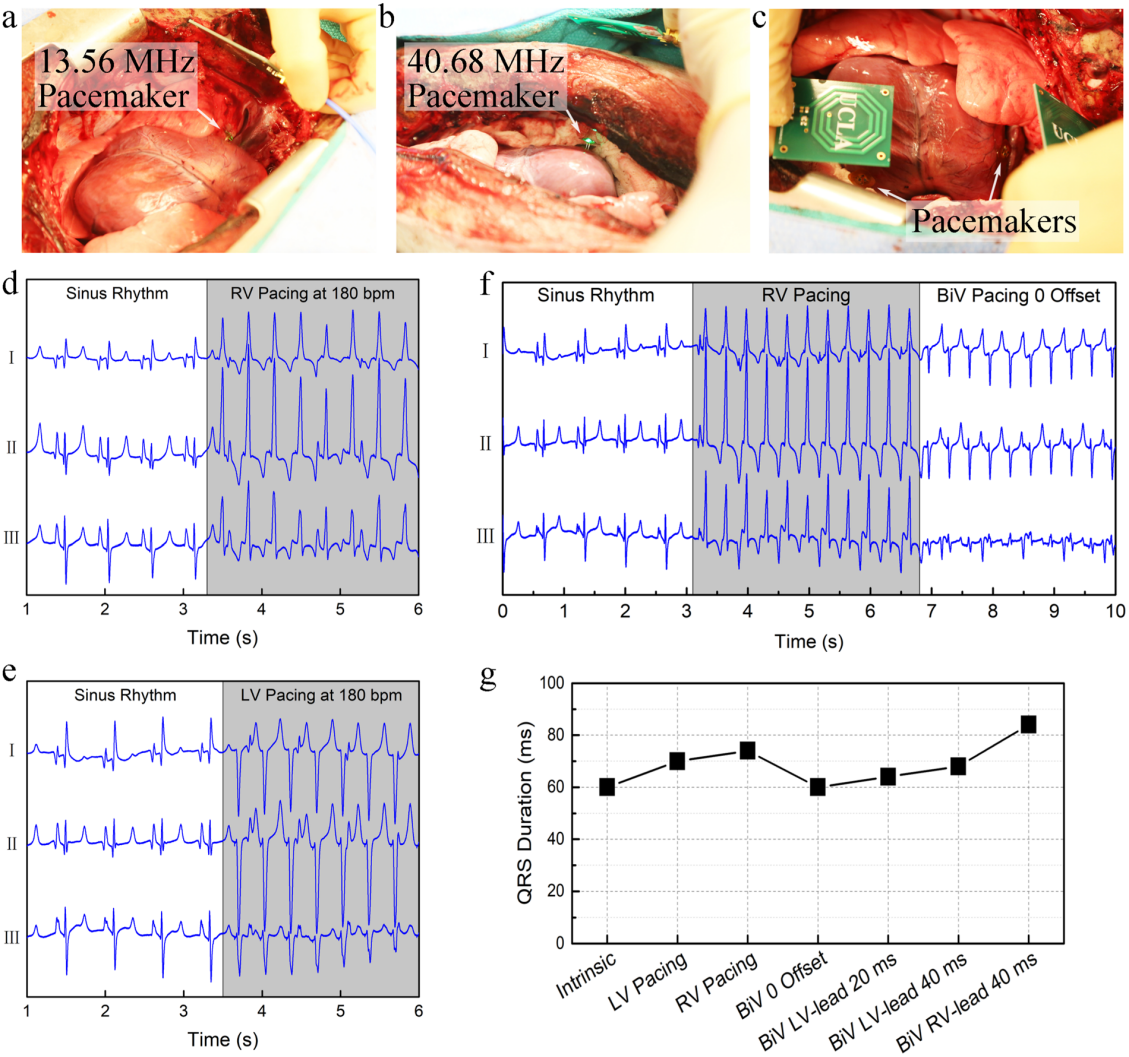
Electrophysiology in the open-chest pacing study. Pictures of open-chest (**a**) RV, (**b**) LV and (**c**) BiV pacing. The ECG recordings showing (**c**) RV and (**d**) LV single-site pacing, and (**f**) a transition from RV single-site pacing to BiV pacing. The QRS durations associated with each pacing modalities are compared in (**g**).

*In vivo* studies were carried out in a porcine model. The 13.56 MHz and 40.68 MHz pacemakers were implanted on the right ventricle (RV) epicardium and left ventricle (LV) epicardium, respectively. The RV and LV single-site pacing are shown in Fig. 4a,b, respectively, and Fig. 4c shows BiV pacing. Pacing was performed at 180 bpm with a pulse width of 0.3 ms in all cases. Figure 4a,b capture the blinking of the LED and, in particular, Fig. 4b shows that the 40.68 MHz pacemaker is powered under the porcine chest from the Tx coil. The electrocardiogram (ECG) of the animal was monitored using the standard 12-lead setup. The ECG recordings of RV and LV single-site pacing are shown in Fig. 4d,e, respectively. RV pacing results in a QRS morphology with an initially positive vector, while LV pacing features a negative initial vector. Most impressively, the ECG tracings of BiV pacing in Fig. 4f shows the transition from the intrinsic sinus rhythm to RV pacing and then to BiV pacing.

To test the impacts of different offsets in BiV pacing, we programmed BiV pacing at multiple offsets between the two ventricles, i.e., LV-lead 20 ms before RV, LV-lead 40 ms before RV, and RV-lead 40 ms before LV. BiV pacing with LV-lead 20 ms and 40 ms are demonstrated in Supplementary Videos S1 and S2 with 10-fold speed reduction. The QRS duration in the ECG tracing has been well used as a prognostic indicator of the clinical effectiveness of synchronized BiV pacing^14,33^. The widening of QRS duration is clinically associated with worsening hemodynamics due to electrical blocks in the conduction system. In this study, the QRS durations corresponding to different single-site and BiV pacing modalities are demonstrated in Supplementary Fig. S3 and the result is summarized in Fig. 4g. The QRS duration of the intrinsic non-pathologic heartbeat is 60 ms and BiV pacing with zero offset renders a similar value. BiV pacing with LV leading by 20 ms and 40 ms also outperforms single-site RV and LV pacing. In contrast, BiV pacing with RV leading by 40 ms shows a worsened result, suggesting a contradiction to the inherent conduction. Diagnostic electrophysiology (EP) catheters (Boston Scientific, MA) inserted into the RV and coronary sinus (CS) successfully measured reproducible changes in myocardial wavefronts associated with each pacing modality as shown in Supplementary Fig. S4a–d. Distinct pacing artifacts with the programmed offsets are also evident.

**Figure 5:**
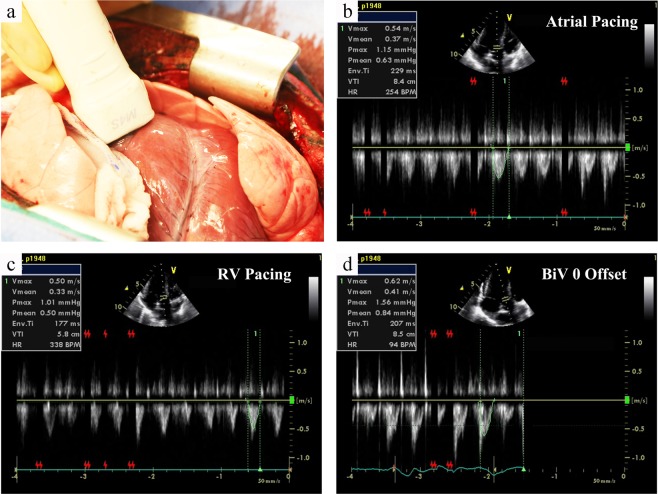
Hemodynamics in the open-chest pacing study. (**a**) Picture of the hemodynamic response test. Pulse wave Doppler images for (**b**) atrial pacing, (**c**) RV pacing and (**d**) BiV pacing with zero offset. The corresponding VTIs are calculated.

Importantly, we further verified the acute benefits of BiV pacing by analyzing the hemodynamic responses. Pulse wave Doppler images were used to calculate the velocity-time integrals (VTI) that correspond to the stroke volumes (SV) in each pacing modality. Aspiration was stopped for a brief period when the echocardiography data were collected as shown in Fig. 5a. The hemodynamics of atrial pacing is first recorded, which renders an inherent VTI of 8.4 cm (Fig. 5b). The VTI during RV pacing, a condition classically associated with increased mortality, shows a significant reduction to 5.8 cm (Fig. 5c). In contrast, BiV pacing with zero offset leads to a VTI close to the inherent value (Fig. 5d), which is consistent with the QRS duration result.

### Closed-chest pacing

**Figure 6:**
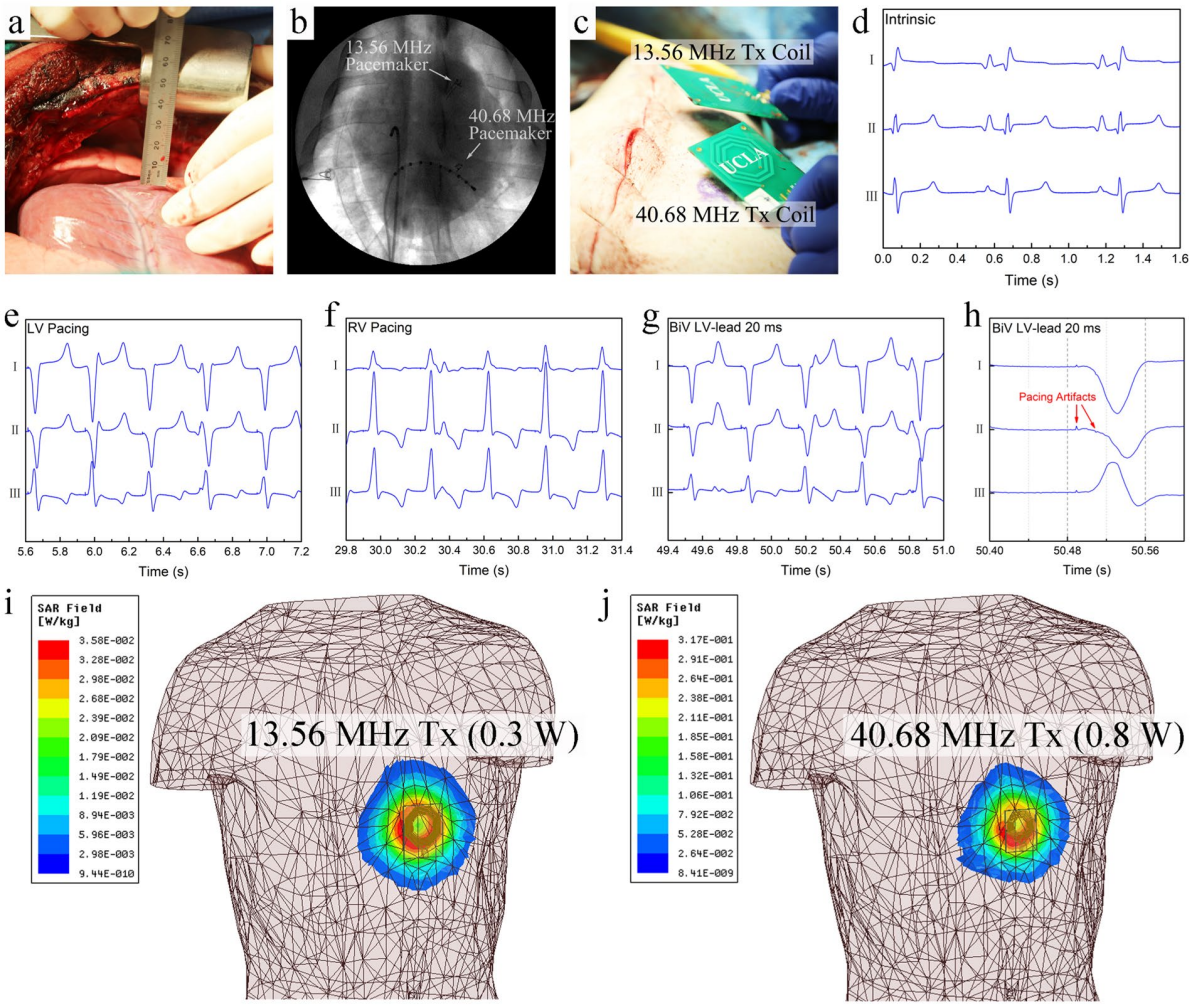
Closed-chest pacing study. (**a**) Picture showing the thickness of the porcine chest. (**b**) Fluorescent image showing the implantation sites of the 13.56 MHz and 40.68 MHz pacemakers after closing the chest. (**c**) Picture showing the position of the corresponding Tx coils outside the chest. ECGs of (**d**) intrinsic heartbeats, (**e**) RV pacing, (**f**) LV pacing, and (**g**) BiV pacing with LV-lead 20 ms. (**h**) QRS waveform of the closed-chest BiV pacing. Simulated SARs associated with the (**i**) 13.56 MHz and (**j**) 40.68 MHz operation settings.

The leadless pacing platform was then verified in a closed-chest setting. The porcine chest is about 5 cm thick so that implanted pacemakers are about 6–8 cm from the body surface as shown in Fig. 6a. The 13.56 MHz and 40.68 MHz pacemakers were implanted on the RV and LV, respectively, with their positions indicated in the fluorescent image in Fig. 6b. Tx coils were placed at approximately 0.5–1 cm outside the sutured chest as shown in Fig. 6c. Closed-chest RV pacing and LV pacing were enabled with Tx power as low as 25 dBm (0.3 W) and 29 dBm (0.8 W), respectively. BiV pacing is also successfully performed in the closed-chest setting as shown in Supplementary Video S3. Figure 6d demonstrates the intrinsic ECGs in the closed-chest setting. The ECGs for RV pacing, LV pacing and BiV pacing with LV-lead 20 ms are shown in Fig. 6e–g, respectively. A section of the QRS waveform during BiV pacing as shown in Fig. 6h evidently shows the pacing artifacts with a programed 20 ms interval.

The SARs associated with the 13.56 MHz and 40.68 MHz links are investigated with the Tx coils positioned at 1 cm proximity to a male torso model. The two links generate the maximum SARs of approximately 0.04 W/kg and 0.3 W/kg, respectively, as shown in Fig. 6i,j, which are significantly lower than the 10 W/kg limit according to IEEE Std C95.1–2005. It is remarkable that unlike prior arts operating at hundreds of MHz^4,6,7^, the proposed inductive coupling links at 13.56 MHz and 40.68 MHz bands render SARs 2–3 orders of magnitude lower than the safety regulation threshold.

## Discussion and Conclusion

The magnetic fields penetrate through tissue, yet decay as the inverse cube of the distance. To harvest the inductive energy over sufficient distance for cardiac pacing, we designed a custom pacemaker IC with significantly reduced power consumption and dedicated power transfer links. The combination of efforts leads to the relative long-distance and reliable operation of the pacemaker. The power transfer links are implemented at 13.56 MHz and 40.68 MHz ISM bands, rendering SARs 2–3 orders of the magnitude below the safety threshold. This strategy can be applied to wirelessly powering many other minimal-power medical implants to realize future in-body sensor networks.

In the pursuit of leadless CRTs, the WiSE CRT system (EBR Systems, Inc.) has recently been launched in a clinical trial^34,35^. The current embodiment of this technology requires the use of a standard dual-chamber pacemaker with only the LV wirelessly paced via ultrasound power transfer, thus limiting the potential benefits of leadless pacing. The implantation of a hardware in the LV endocardium also necessitates a retroaortic access coursing through the arterial system. Because the pacing lead is placed on the LV endocardium, antiplatelet therapy is warranted. Our technology, however, does not have such limitations. The proposed work demonstrates synchronized leadless pacing over multiple sites for the first time, and this approach can potentially offer more flexibility and advantages in customizing CRTs for different individuals. In the comparison of the wireless powering strategies, magnetic fields in inductively coupling systems are not affected by biological tissues, while ultrasound power transfer essentially works through vibrations, which could deteriorate over air-filled viscera and obstructions, such as lungs and bones^36^.

The current form factor of the pacemaker is designed for epicardial pacing, which, for example, is a typical clinical approach for treating pediatric patients with congenital heart disease and requiring open-chest placement of epicardial pacing leads^37^. Our on-going research focuses on the miniaturization of the device and the associated venous delivery technologies, for example, epicardial implantation via venous tributaries that eliminates the necessity for anticoagulation. Future iterations of the IC will also incorporate a unique identification code to avoid false-triggering.

In summary, we report wirelessly powered pacemakers which feature µW-level power consumption and a miniaturized and flexible form factor that is suitable for epicardial implantation. Two pacemakers were implemented at 13.56 MHz and 40.68 MHz ISM bands, demonstrating the maximum operating distance of 11 cm and 8.5 cm from 1 W Tx coils, respectively. They were implanted on the RV and LV of a porcine model, respectively, which successfully demonstrated the leadless CRT. Different pacing modalities, i.e., single-site pacing and BiV pacing with different offsets, were studied, and BiV pacing showed improvements in the QRS duration and cardiac stroke volumes. The closed-chest leadless BiV pacing was further validated. The 13.56 MHz and 40.68 MHz links only required the Tx power of 0.3 W and 0.8 W, respectively, which lead to SARs 2–3 orders of magnitude below the safety threshold. The proposed batteryless and leadless pacing platform shows promise in providing advanced therapies in cardiac synchronization.

## Methods

### Electromagnetic simulations

The inductively coupling link is simulated with Maxwell (Ansys Inc.) and Simplorer (Ansys Inc.). The SAR is simulated with HFSS (Ansys Inc.)

### Equipment setup

According to the circuit schematic in Fig. 3h, the arbitrary waveform generator (33500B, Keysight Inc.) outputs two duty-cycled pulse signals that modulate two signal sources (E4431B and E4432B, Hewlett Packard Inc.) via pulse modulation. The 13.56 MHz and 40.68 MHz RF signals are amplified by two following power amplifiers (ZHL-20W-13 + , Mini-Circuits Inc.). The gain of the power amplifier is 50 dB.

### *In vivo* study

*In vivo* studies were carried out in a porcine model (53 kg, Yorkshire, Female, n = 1). All experiments and methods were performed in accordance with the protocols approved by the Institutional Animal Care and Use Committee (IACUC) of Texas Heart Institute. The animal was anesthetized using isoflurane anesthesia (0.5–3.0%) prior to the study. Diagnostic electrophysiology (EP) catheters (Boston Scientific, MA) were inserted into the RV and CS for continuously monitoring intracardiac signals during the study. The vitals of the animals were also monitored using a standard 12-lead ECG setup. The EP catheters and surface ECG pads were connected to LABSYSTEM PRO EP Recording system (Boston Scientific, MA) for collecting and storing EP data. Hemodynamic data were collected using a GE VIVID 7 ultrasound machine (GE Healthcare, IL). Aspiration was stopped for a brief period when the echocardiography data was collected. The animal was euthanized at the end of the study humanely as per American Veterinary Medical Association (AVMA) guidelines.

## Supplementary information

Supplementary Information.

Video S1.

Video S2.

Video S3.

## Data Availability

All data generated or analyzed during this study are available from the corresponding author upon request.
