# Author Correction: Synchronized Biventricular Heart Pacing in a Closed-chest Porcine Model based on Wirelessly Powered Leadless Pacemakers

**DOI:** 10.1038/s41598-020-63288-x

**Published:** 2020-04-07

**Authors:** Hongming Lyu, Mathews John, David Burkland, Brian Greet, Allison Post, Aydin Babakhani, Mehdi Razavi

**Affiliations:** 1 Electrical and Computer Engineering Department, University of California Los Angeles, 420 Westwood Plaza, Los Angeles, CA 90095 USA; 2 Texas Heart Institute, 6770 Bertner Avenue, Houston, TX 77030 USA; 3 School of Medicine, Baylor College of Medicine, 1 Baylor Plaza, Houston, TX 77030 USA

Correction to: *Scientific Reports* 10.1038/s41598-020-59017-z, published online 07 February 2020

This Article contains a typographical error in the Results section, under subheading ‘Pacemaker design’ where,

“Adding a C_tune_ of 82 µF in parallel with Rx coil tunes the SRF to be at around 13.56 MHz ISM band.”

should read:

“Adding a C_tune_ of 82 pF in parallel with Rx coil tunes the SRF to be at around 13.56 MHz ISM band.”

Additionally, the Acknowledgements section in this Article is incomplete.

“We thank Dr. B. Aazhang and Dr. J. Cavallaro for helpful discussions.”

should read:

"The animal research was supported by the Sultan Qaboos Chair in Cardiology at the St. Luke’s Foundation and the device development was supported by UCLA internal funding. We also thank Dr. B. Aazhang and Dr. J. Cavallaro for helpful discussions."

