# Supplementary material for: Synchronized Biventricular Heart Pacing in a Closed-chest Porcine Model based on Wirelessly Powered Leadless Pacemakers
Source: Sci Rep. 2020 Feb 7;10:2067. doi: 10.1038/s41598-020-59017-z (PMC7005712; doi:10.1038/s41598-020-59017-z)
Supplement: Supplementary file 1 — Supplementary Information. [file 41598_2020_59017_MOESM1_ESM.docx]

**Supplementary Information:**

**Synchronized Biventricular Heart Pacing in a Closed-chest Porcine Model based on Wirelessly Powered Leadless Pacemakers**

Hongming Lyu^1^, Mathews John^2^, David Burkland^3^, Brian Greet^3^, Allison Post^2^, Aydin Babakhani^1^*, Mehdi Razavi^2,3^*

1. Electrical and Computer Engineering Department, University of California Los Angeles, 420 Westwood Plaza, Los Angeles, CA 90095, USA
2. Texas Heart Institute, 6770 Bertner Avenue, Houston, TX 77030, USA
3. School of Medicine, Baylor College of Medicine, 1 Baylor Plaza, Houston, TX 77030, USA

Corresponding Authors:

Aydin Babakhani: aydinbabakhani@ucla.edu

Mehdi Razavi: [razavi@bcm.edu](mailto:razavi@bcm.edu)


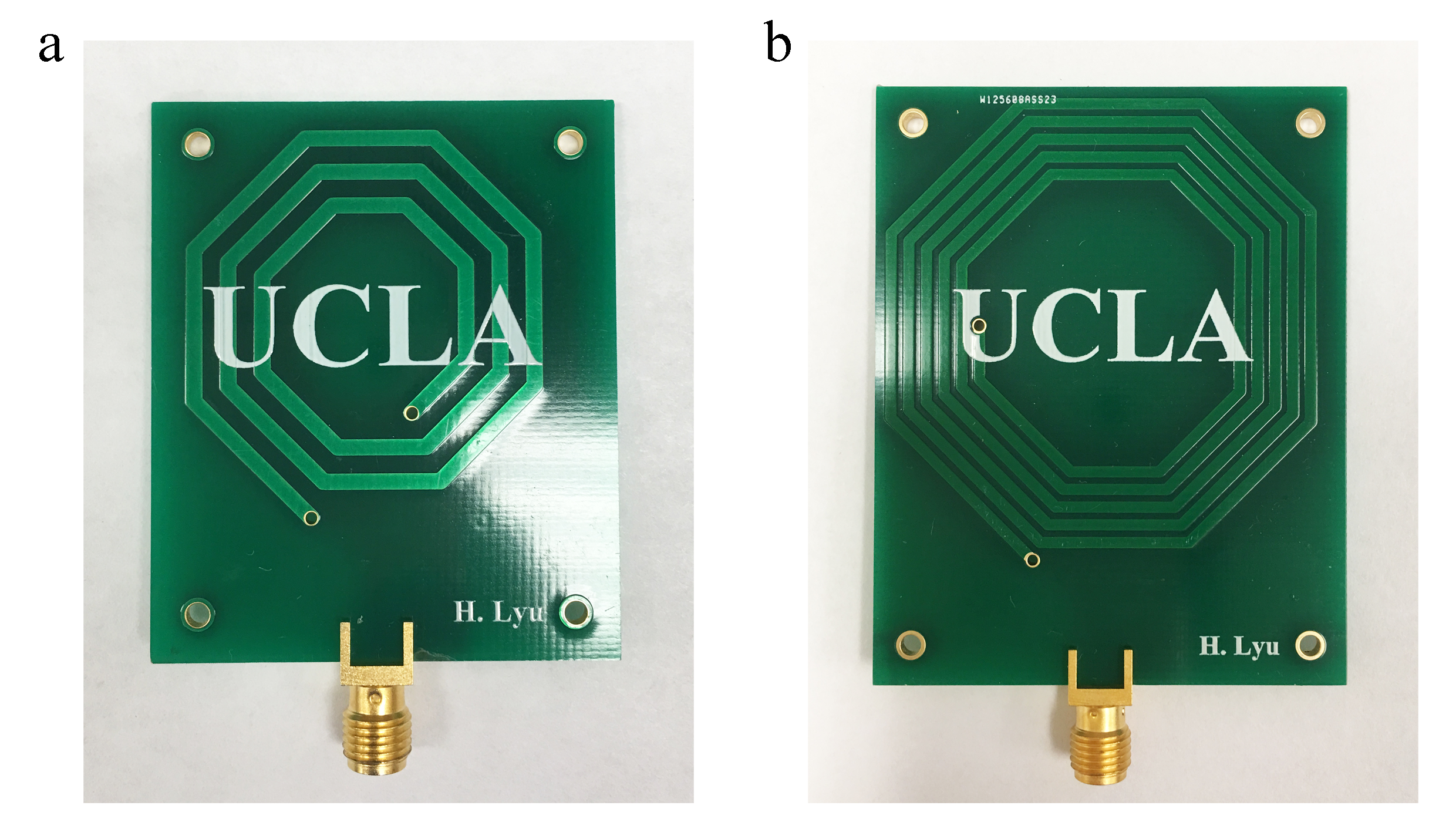


Fig. S1. Photos of the backside of the Tx coils. The backside of (a) the 40.68 MHz Tx coil and (b) the 13.56 MHz Tx coil.


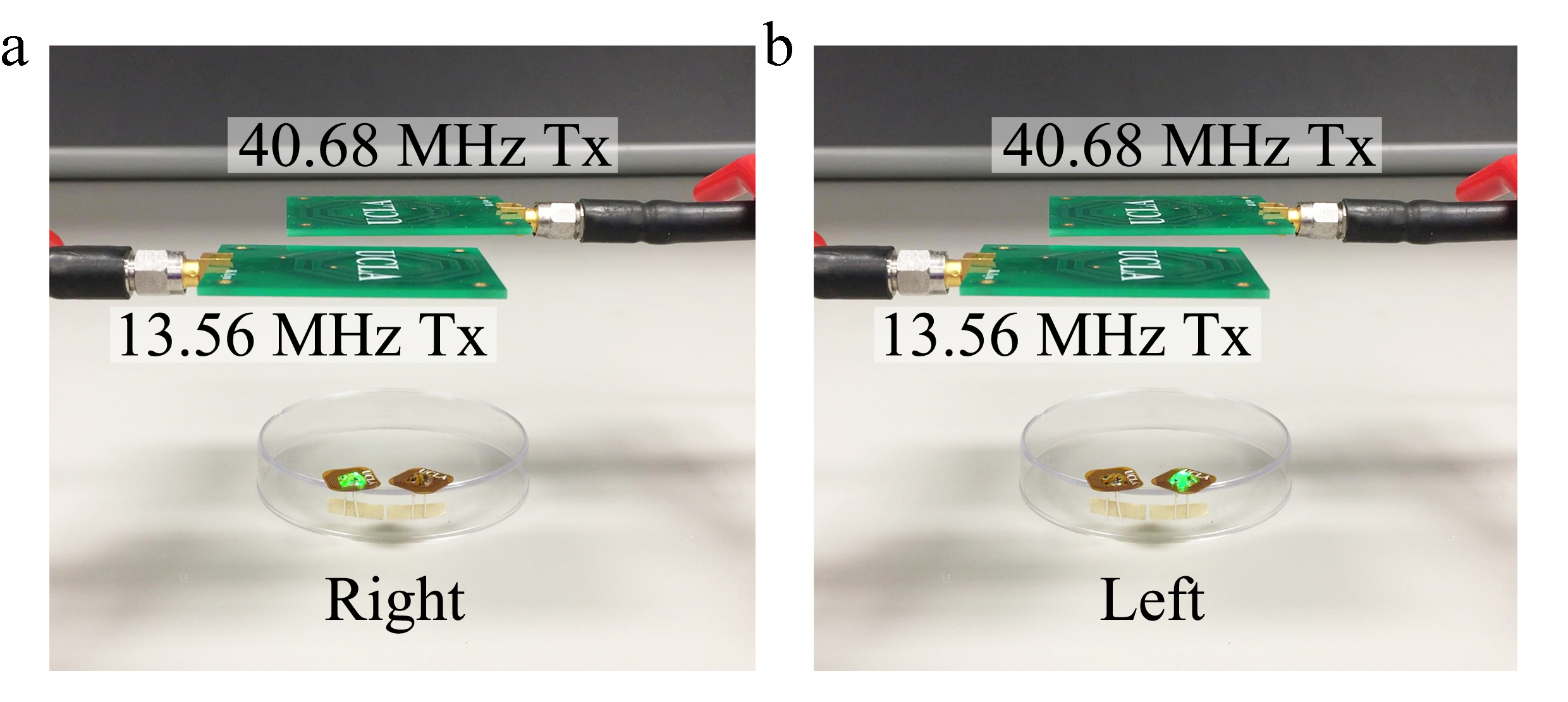


Fig. S2. Photos illustrating the independent control of a 13.56 MHz pacemaker and a 40.68 MHz pacemaker in close proximity. (a) 13.56 MHz pacemaker is selectively controlled by the 13.56 MHz Tx coil, while (b) 40.68 MHz pacemaker is selectively controlled by the 40.68 MHz Tx coil. Both links are in operation.


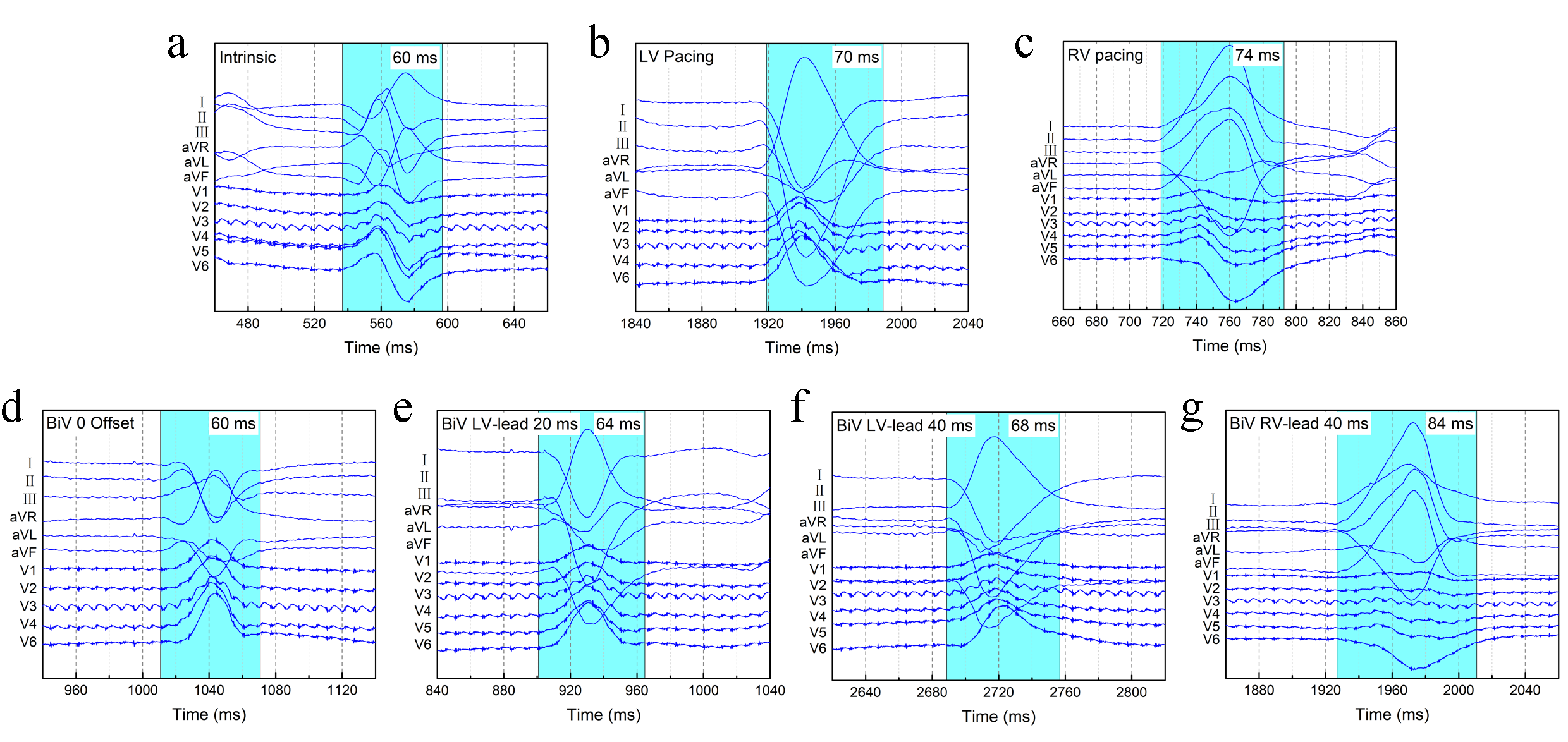


Fig. S3. The QRS waveforms of different pacing modalities: (a) intrinsic heartbeat, (b) LV pacing, (c) RV pacing, (d) BiV pacing with zero offset, (e) BiV pacing with LV-lead 20 ms, (f) BiV pacing with LV-lead 40 ms, (g) BiV pacing with RV-lead 40 ms.


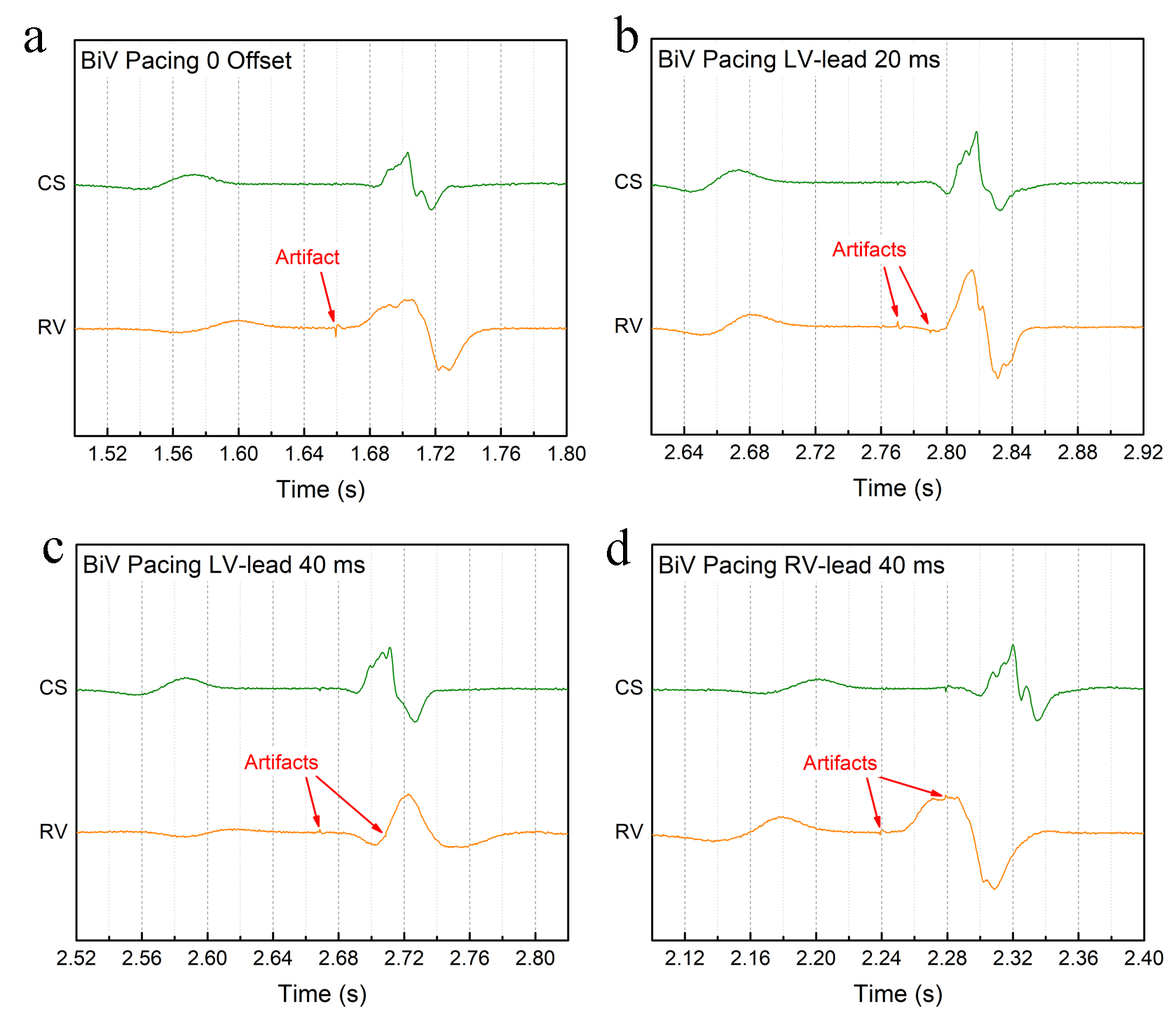


Fig. S4. The intracardial recordings of BiV pacing with (a) zero offset, (b) LV-lead 20 ms, (c) LV-lead 40 ms, and (d) RV-lead 40 ms showing different myocardial wavefronts.
